# Determination of Supplier-to-Supplier and Lot-to-Lot Variability in Glycation of Recombinant Human Serum Albumin Expressed in *Oryza sativa*

**DOI:** 10.1371/journal.pone.0109893

**Published:** 2014-10-09

**Authors:** Grant E. Frahm, Daryl G. S. Smith, Anita Kane, Barry Lorbetskie, Terry D. Cyr, Michel Girard, Michael J. W. Johnston

**Affiliations:** Centre for Biologics Evaluation, Biologics and Genetic Therapies Directorate, Health Canada, Ottawa, Ontario, Canada; Russian Academy of Sciences, Institute for Biological Instrumentation, Russian Federation

## Abstract

The use of different expression systems to produce the same recombinant human protein can result in expression-dependent chemical modifications (CMs) leading to variability of structure, stability and immunogenicity. Of particular interest are recombinant human proteins expressed in plant-based systems, which have shown particularly high CM variability. In studies presented here, recombinant human serum albumins (rHSA) produced in *Oryza sativa* (Asian rice) (OsrHSA) from a number of suppliers have been extensively characterized and compared to plasma-derived HSA (pHSA) and rHSA expressed in yeast (*Pichia pastoris* and *Saccharomyces cerevisiae*). The heterogeneity of each sample was evaluated using size exclusion chromatography (SEC), reversed-phase high-performance liquid chromatography (RP-HPLC) and capillary electrophoresis (CE). Modifications of the samples were identified by liquid chromatography-mass spectrometry (LC-MS). The secondary and tertiary structure of the albumin samples were assessed with far U/V circular dichroism spectropolarimetry (far U/V CD) and fluorescence spectroscopy, respectively. Far U/V CD and fluorescence analyses were also used to assess thermal stability and drug binding. High molecular weight aggregates in OsrHSA samples were detected with SEC and supplier-to-supplier variability and, more critically, lot-to-lot variability in one manufactures supplied products were identified. LC-MS analysis identified a greater number of hexose-glycated arginine and lysine residues on OsrHSA compared to pHSA or rHSA expressed in yeast. This analysis also showed supplier-to-supplier and lot-to-lot variability in the degree of glycation at specific lysine and arginine residues for OsrHSA. Both the number of glycated residues and the degree of glycation correlated positively with the quantity of non-monomeric species and the chromatographic profiles of the samples. Tertiary structural changes were observed for most OsrHSA samples which correlated well with the degree of arginine/lysine glycation. The extensive glycation of OsrHSA from multiple suppliers may have further implications for the use of OsrHSA as a therapeutic product.

## Introduction

Human serum albumin (HSA) is the most abundant protein component of human plasma. Due to its abundance and specific properties, it is utilized for numerous *in vitro* studies and pharmaceutical applications [1]. For instance, we and others have used albumin to investigate the interactions of a model blood protein with nanomaterials [2], [3] and as a model for induced drug release from nanoscale drug delivery systems [4]. HSA has been used for the treatment of hypoalbuminemia due to severe burns (up to 10 g/dose) [5], [6] and for chronic liver cirrhosis, and has been proposed as a treatment for Alzheimer's disease [7]. This protein has also been used in nanoscale drug delivery systems such as Abraxane (130 nm albumin nanoparticle for the delivery of Paclitaxel) and Albuferon (an interferon α-2b/albumin fusion protein) [8], [9]. The complex nature of the protein's surface also allows it to function as an excipient, preventing protein aggregation and adsorption to glass vials [10], [11].

Obtaining HSA from human plasma has raised concerns about the possible transmission of infectious agents resulting in reduced use of plasma HSA (pHSA) as a drug excipient [5], [6]. These fears, as well as supply issues [6], have spawned the development of recombinant versions of the protein from a number of expression systems including yeast (*Pichia pastoris* and *Saccharomyces cerevisiae*) and Asian rice (*Oryza sativa*). One recombinant HSA (rHSA) has been approved by the FDA and EMA for the production of MMR™ II childhood vaccine and it is conceivable that manufacturers will seek approval to use rHSA in additional therapeutic and vaccine formulations.

Recently, our group has investigated the interaction of 1,2-dipalmitoyl-*sn*-glycero-3-phosphocholine (DPPC) liposomes with two rHSAs produced from different expression systems (1). We noted dramatic differences in the thermal stability between rHSA expressed in *Pichia pastoris* (PprHSA) and *Oryza sativa* (OsrHSA) [3]. OsrHSA showed considerably higher thermal stability than PprHSA, which we attributed to either the presence of stabilizing fatty acids (FA) or the numerous hexose modifications on lysine and arginine residues that were identified [3]. The presence of either bound ligands or chemical modifications (CMs) on rHSA is of particular interest, as previous studies have shown the presence of FA or glycation can alter structure, improve thermal stability, and alter ligand binding to albumin [12]–[16].

Due to the broad use of albumin as a therapeutic and in pharmaceutical research, as well as the growing popularity of *O. sativa* as a cost-efficient [17], high yield [18] expression system, we believe it is important to determine if the OsrHSA CMs and bound FAs are inherent to the production process and if they alter the protein's drug binding properties. If so, there are important implications for the viability of rice as an expression system for rHSA and possibly other recombinant proteins. In addition to the production of albumin, rice is used, or has been proposed as an expression system for recombinant human transferrin, human growth hormone and the envelope protein of Japanese encephalitis virus [17], [18].

To this end we sourced commercially available rHSAs expressed in yeast (*P. pastoris*, and *S. cerevisiae*): an rHSA approved for use in the production of MMR II™ vaccine (Recombumin®, expressed in *S. cerevisiae)*, a commercially available plasma-derived HSA (pHSA) and four rHSAs expressed in *O. sativa* from different suppliers. These samples were then subjected to an extensive array of biophysical analyses. These analyses showed that rHSA expressed in *O. sativa* generally displayed greater heterogeneity, higher quantities of non-monomeric species, greater numbers of glycated residues and greater degree of glycation of those residues. We also observed a positive correlation between the numbers of glycated residues/degree of glycation of OsrHSA and alterations to tertiary structure.

## Materials and Methods

### Materials

Chemicals, essentially FA-free pHSA (A3872 Lot#090M7001V, ≥99% purity), recombinant human serum albumin expressed in *Saccharomyces cerevisiae* (ScrHSA, Lot# SLBD2407, ≥99% purity, Albucult), *Oryza sativa* [OsrHSA, Lot# SLBC7527V (OsrHSA-sig-C), Lot# SLBG7405V (OsrHSA-sig-G), Lot# SLBH9636V (OsrHSA-sig-H) and Lot# SLBJ1196V (OsrHSA-sig-J) 100% purity, Cellastim] and *Pichia pastoris* (PprHSA, Lot# 080M1580V, ≥99% purity, Albagen) were sourced from Sigma-Aldrich (St. Louis, MO, USA). OsrHSA was also obtained from eEnzyme LLC (Gaithersburg, MD, USA) (OsrHSA-phy) (Lot# 20130110, >99% purity, Phyto-HSA), ScienCell Research Laboratories (Carlsbad, CA, USA) (OsrHSA-sci) (Lot# BJABAA42, ≥99% purity, Oryzogen) and amsbio LLC (Cambridge, MA, USA) (OsrHSA-ams) (Lot #20101008, >95% purity, ecoHSA). Recombumin (Lot# PDP100106) was donated by Novozymes Biopharma (Cambridge, MA, USA) or purchased from a local pharmacy. Amicon Ultra 0.5 mL 3000 Da molecular weight cut-off (MWCO) centrifugal filter devices were purchased from Millipore (Billerica, MA, USA). Trypsin and chymotrypsin were Promega sequencing grade purchased from Fisher Scientific Canada (Ottawa, ON, Canada). Vivacon 500 10 kDa molecular weight cut-off filters were from Sartorius Stedim Biotech North America (Bohemia, NY, USA).

### Albumin sample preparation

Albumin samples were prepared as described previously [3], [19]. Briefly, samples were buffer exchanged into 5 mM sodium phosphate (pH 7.4), with Amicon Ultra 0.5 ml 3000 Da MWCO centrifugal filter devices after pre-rinsing the filters with buffer. Protein concentrations were measured using a BCA assay kit (Sigma-Aldrich, St. Louis, MO, USA). Protein integrity after buffer exchange was assessed with 1-D SDS−PAGE using SYPRO Ruby protein stain (Molecular Probes, Eugene, OR, USA) and a Bio-Rad Molecular Imager Gel Doc XR+ system with Quantity One 1-D analysis software according to the manufacturer's instructions (Bio-Rad, Mississauga, ON, Canada).

### Size Exclusion Chromatography (SEC)

The size-exclusion chromatography system was a Waters Alliance 2695 Separations Module fitted with a Waters 2996 Photodiode Array Detector (Waters Corporation, Milford, MA, USA). Instrument operation and data acquisition and manipulation were carried out with Waters Empower 2 Chromatography Manager (Waters Corporation). A YMC-Pack Diol-200 column (Product# DL20S05-5008WT, YMC America, Inc., Allentown, PA, USA) with internal dimensions of 500×8.0 mm was used at a flow rate of 0.8 ml/min. The mobile phase consisted of 0.1 M sodium phosphate, 0.15 M sodium chloride (pH 7.0) and peaks were detected at a wavelength of 214 nm.

### Capillary Electrophoresis (CE)

The capillary electrophoresis (CE) system was a PACE MDQ (Beckman Coulter Canada Inc., Mississauga, ON, Canada) fitted with a variable wavelength ultraviolet (UV)–visible detector. Instrument operation and data acquisition and manipulation were carried out with 32Karat software (Beckman Coulter). Fused silica capillaries were from Polymicro Technologies (Phoenix, AZ, USA) and were held in cassettes with an 800 µm aperture. Analyses were carried out under conditions described previously [20]. Briefly, separations were performed in capillaries, 50 µm i.d. × 60 cm in length (50 cm effective length) using 20 mM disodium hydrogen phosphate containing 5 mM 1,4-diaminobutane and adjusted to pH 8.5 as background electrolyte (BGE). New capillaries were first conditioned by consecutive rinses with 1 M HCl, water, 1% NaOH and BGE. A final conditioning step was with a 1 mg/mL pHSA solution for 30 min. At the end of each injection, reconditioning of the capillary was performed with a sequence of rinses consisting of 1 M HCl for 3 min, water for 3 min, 1% NaOH for 3 min, water for 3 min and buffer for 3 min. Samples were injected by pressure for 10 s before applying a constant voltage of 25 kV and monitoring at 200 nm for 60 minutes and maintaining the capillary temperature at 25°C.

### Reversed Phase High-Performance Liquid Chromatography (RP-HPLC)

The HPLC system consisted of a Waters Alliance 2695 chromatograph equipped with a column heater and an autosampler with a sample cooling device, coupled to a Waters 2996 U/V–Vis photodiode array detector. Data acquisition and integration were performed with Empower Pro Software from Waters. Separation conditions were as described previously [21]. Briefly, the column was an Aquapore RP-300, C8, 7 µm, 220×2.1 mm i.d. (Brownlee) and was maintained at 50°C. Mobile Phase A (MP A) consisted of 0.05% trifluoroacetic acid (TFA) in 10% acetonitrile/90% water; Mobile Phase B (MP B) was 0.05% TFA in 90% acetonitrile/10% water; Mobile Phase C (MP C) was 0.05% TFA in acetonitrile. The column was equilibrated with a mixture of MP A and MP B (70∶30) until a stable baseline was obtained. Elution was carried out using a multi-step gradient consisting of MP A/MP B (70∶30) for 1 min (at 0.7 ml/min), linear gradient to MP A/MP B (65∶35) over 5 min (at 0.7 ml/min), linear gradient to MP A/MP B (61∶39) over 19 min (at 0.7 mL/min), linear gradient to MP A/MP B (50∶50) over 10 min (at 0.7 mL/min), linear gradient to MP C over 5 min (at 1.0 mL/min), MP C for 12 min, linear gradient to MP A/MP B (70∶30) over 3 min. The effluent was monitored at 220 nm.

### Liquid Chromatography–Mass Spectrometry (LC-MS) Analysis - Sample Preparation

For each of the rHSA samples, approximately 10 µg were diluted in 50 mM ammonium bicarbonate to a total volume of 200 µl. To each solution 10 µl of 250 mM dithiothreitol (DTT) in 50 mM NH_4_HCO_3_ were added followed by incubation for 1 hour at 60°C. Next, 20 µl of 250 mM iodoacetamide in 50 mM NH_4_HCO_3_ were added and samples were incubated at room temperature in the dark for 30 min. The alkylation reactions were quenched by the addition of an additional 20 µl of 250 mM DTT and the samples were split into 2×125 µl aliquots and transferred onto 10 kDa MWCO spin filters. Samples were centrifuged for 20 min at 14,000×g then washed with 2×200 µl of 50 mM NH_4_HCO_3_ in the same manner.

Trypsin and chymotrypsin solutions were prepared by adding 1000 µl of 50 mM NH_4_HCO_3_ to 20 µg and 25 µg lyophilized protein, respectively. One 125 µl aliquot from each sample was digested with trypsin and the other with chymotrypsin. Digestion was carried out by spinning 100 µl of trypsin solution through the filters at 10,000×g over 20 min, then spinning 100 µl of 50 mM NH_4_HCO_3_ through the filter under the same conditions. The flow through from the digestion steps was collected in clean tubes and evaporated to dryness in a vacuum centrifuge then re-suspended in 40 µl of injection solvent (3% acetonitrile, 0.2% formic acid and 0.05% TFA in water) prior to LC-MS/MS analysis.

### LC-MS Analysis - Sample Analysis

For each sample, triplicate 2 µl aliquots were analyzed by loading onto a Waters Symmetry C18 trap column (180 µm x 20 mm with 5 µm beads) and desalting with 0.1% formic acid in water (solvent A) for 3 min at 5.0 µl/min before separating on a Waters nanoAcquity UPLC BEH130 C18 reverse phase analytical column (100 µm x 100 mm with 1.7 µm beads). Chromatographic separation was achieved at a flow rate of 0.500 µl/min over 70 min in six linear steps as follows (solvent B was 0.1% formic acid in acetonitrile): Initial – 3% B, 2 min – 10% B, 40 min – 30% B, 50 min – 95% B, 55 min – 95% B, 56 min – 3% B, final – 3% B. The eluting peptides were analyzed by MS and MS/MS using a Waters Synapt HDMS system operating in data dependant acquisition (DDA) mode. MS survey scans were 1 s in duration and MS/MS data were collected on the four most abundant peaks until either the total ion count exceeded 3000 or until 3 s elapsed. Within each analysis, redundant analyses were limited by excluding selected peaks ±1.15 mass-to-charge (m/z) for 60 s. Between triplicate analyses, previously selected peaks were prevented from being reanalyzed by using m/z (±1.15) and retention time (±60 s) as exclusion criteria. Peaks from singly-charged peptides were also excluded from selection for MS/MS analysis. The instrument was calibrated prior to sample analysis using the fragmentation products of [Glu1]-Fibrinopeptide B. Calibration accuracy was maintained throughout the analyses using a nano-lock spray of 100 fmol/µl [Glu1]-Fibrinopeptide B, which was sampled for 1 s once every 30 s. The lock mass correction was applied to the data during processing.

### LC-MS Analysis - Qualitative Data Processing

Data were qualitatively analyzed using the Mascot software package, available from Matrix Science Ltd. (Boston, MA, U.S.A.). The raw data were processed using Mascot Distiller (version 2.4.2) to create Mascot Generic Files (MGFs) and database searches were performed using Mascot (version 2.4), against the human protein entries in the 2013_01 UniProtKB/Swiss-Prot database. MGF files from the triplicate analyses of both the trypsin and chymotrypsin digests were combined and submitted as a single search for each sample. Peptide and MS/MS mass tolerances were 100 ppm and 0.1 Da, respectively, and semi-tryptic and semi-chymotryptic peptides, from 2+ to 5+ charge state and having up to three missed cleavages, were considered. Carbamidomethylation of cysteine was specified as a fixed modification and oxidation of methionine and hexose addition on lysine and arginine were considered as variable modifications. The mass spectrometry proteomics data have been deposited to the ProteomeXchange Consortium via the PRIDE partner repository with the dataset identifier PXD001248 and DOI 10.6019/PXD001248.

### LC-MS Analysis - Quantitative Data Processing

Quantitative data analysis was performed using Progenesis LC-MS software (v4.1) available from Nonlinear Dynamics (Newcastle, UK). The raw data from the triplicate LC-MS analyses of the trypsin digests from each of the albumin samples were imported to Progenesis LC-MS and were aligned and normalized on all peaks using default parameters. The Progenesis peak picking algorithm then created 3-dimensional contour plots from each file and identified the contained ‘features’ (e.g., peptide peaks). Progenesis then created a single MGF file from the processed data from all samples, which was submitted to Mascot for database searching using the parameters noted earlier. Mascot search results were exported in.xml format and imported back into Progenesis allowing peptide identifications to be assigned to many of the features. All features identified as hexose-modified peptides were manually examined and adjusted if necessary to ensure peaks were accurately delineated and contained minimal signal from overlapping features and/or instrument background signal. Comparison of relative peak intensities of features identified as hexose modified peptides allowed for relative quantification at specific residues.

### Circular Dichroism Spectropolarimetry

Circular dichroism spectropolarimetry (far U/V CD) analysis of albumin samples was conducted as described previously [3], [19]. Albumin samples were diluted to 0.15 mg/ml with buffer (5 mM sodium phosphate, pH 7.4) and analyzed with a Jasco 815 spectropolarimeter (Jasco International Co., Ltd. Tokyo, Japan) equipped with a Peltier thermal control unit set to room temperature (22°C) in 1 mm quartz cuvettes (Hellma, Müllheim, Germany). The instrument and thermal control unit were controlled with Spectra Manager Software (Jasco International Co.). Each far U/V CD spectrum for secondary structure analysis represents the average of 5 scans from 260 to 190 nm with a data pitch of 1 nm and a response time of 1 s. Spectra were corrected for buffer. The calculation of secondary structure was conducted using Dichroweb (http://dichroweb.cryst.bbk.ac.uk/html/home.shtml) with the CDSSTR algorithm [22] utilizing reference set 4 (190-240 nm) with results presented as mean +/− standard deviation of 3 separate experiments.

Thermal denaturation studies were carried out by monitoring CD (millidegrees) at 222 nm between 20°C and 90°C in increments of 2°C per minute. Due to the predominant alpha helical nature of HSA, monitoring the CD minimum at 222 nm - a characteristic of alpha helical structure - is ideal for assessing the thermal stability of albumin and other predominantly alpha helical proteins [3], [19], [23]. Samples were prepared in 1 mm quartz cuvettes. Fractional (normalized) change was calculated according to previously published studies, using the following formula:

F(obs)  =  [Eobs(T) – Emax]/[E20-Emax]

where Eobs(T) is the ellipticity at 222 nm at temperature T, Emax is the ellipticity at the maximum temperature (°C) used, and E20 is the ellipticity at the initial temperature of 20°C [24].

### Fluorescence Spectroscopy Analysis

Intrinsic protein fluorescence was measured to assess the structure of each of the albumin samples as it is highly sensitive to secondary/tertiary structural changes. Analysis of albumin in 5 mM sodium phosphate buffer (pH 7.4) was conducted with a Varian Cary Eclipse Fluorescence Spectrometer at room temperature controlled with Cary Eclipse Scan Application software (Agilent Technologies, Mississauga, ON, Canada). Scans were conducted with an excitation wavelength of 280 nm and emission wavelengths of 290 to 500 nm with excitation/emission slit widths of 5 nm. Fluorescence spectra were corrected for buffer and presented as mean values, +/− standard deviations of at least three separate experiments. It should be noted that >95% of HSA's intrinsic fluorescence is attributed to the single tryptophan residue at position 214 [25].

### Differential Scanning Calorimetry

Albumin samples in 5 mM sodium phosphate buffer (pH 7.4) were degassed and analyzed with a Calorimetry Sciences Corporation N-DSC III controlled with DSCRun software (now TA Instruments, New Castle, Delaware, USA). Samples were run under 3 atmospheres of pressure with the temperature increasing from 15°C to 110°C at a rate of 2°C per minute. Data were analyzed with Calorimetry Sciences Corporation CPCalc software with the transition temperature (Tm) presented as mean values, +/− standard deviations of at least three separate experiments.

### Drug Binding Analysis

Fluorescence quenching has been effectively used to assess drug binding to albumin [15]. Acetylsalicylic acid (ASA) and HSA in 5 mM sodium phosphate buffer (pH 7.4) were mixed at drug:protein molar ratios between 1 and 20 and the intrinsic fluorescence assessed as noted above. The maximum tryptophan fluorescence intensity for each protein/drug sample was used to calculate binding constants and number of bound drug molecules for each sample [26], [27].

### Defatting OsrHSA

OsrHSA-sig-C was rapidly defatted using published procedures [28]. Briefly, OsrHSA was exchanged into 5 mM citrate buffer (pH 3.2) as described above in “Albumin sample preparation” to partially unfold the protein to allow for improved removal of bound FAs. OsrHSA was applied to a 4 ml column packed with long chain (C13–C18) alkyl ethers-substituted hydroxyalkoxypropyl-dextran (Sigma-Aldrich) equilibrated with citrate buffer (pH 3.2) at a flow fate of 0.5 ml/min run on a GE Healthcare ÄKTApurifier (Piscataway, NJ, USA). The column and all buffer solutions were maintained at room temperature. Protein was eluted from the column with 5 mM citrate buffer (pH 3.2) and the column was regenerated with 10 column volumes of methanol. Defatted OsrHSA-sig-C (dfOsrHSA-sig-C) was then buffer exchanged into 5 mM sodium phosphate (pH 7.4) immediately after elution from the column. DfOsrHSA-sig-C was examined by SDS-PAGE and size-exclusion chromatography as described above.

## Results and Discussion

As a consequence of our recent studies showing marked differences between two rHSA expressed in yeast and Asian rice with respect to hexose modification of lysine and arginine residues, thermal stabilities and interactions with liposomal membranes [3], a more complex biophysical investigation involving commercially available OsrHSA from four suppliers with four lots of OsrHSA from a single supplier (Sigma-Aldrich) was carried out.

### SDS-PAGE Analysis of rHSA Products

Initially, all albumin samples were assessed by SDS-PAGE and SYPRO ruby staining. All albumins showed major bands at approximately 65 kDa, similar to the published molecular weight for HSA of 66.5 kDa [6] (Figure S1). To provide a higher resolution examination of potential pHSA and rHSA heterogeneity, the albumin samples were further analyzed by SEC, RP-HPLC and CE.

### Analysis of rHSA Products by SEC, CE and RP-HPLC

**Figure 1:**
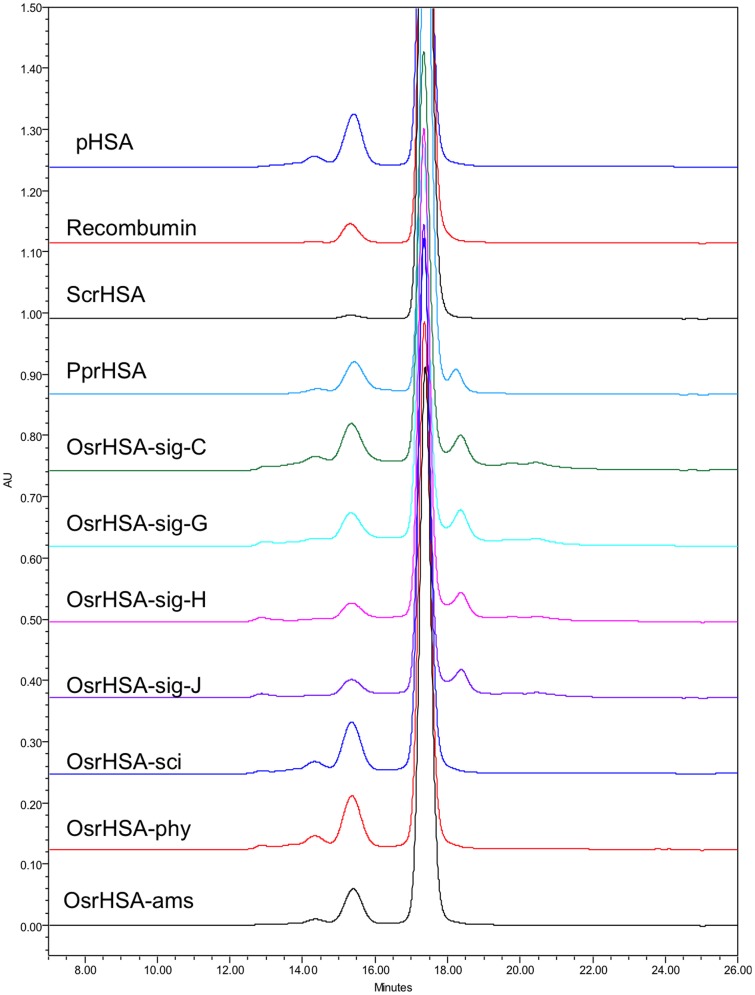
Representative chromatograms of pHSA and rHSA products obtained by SEC utilizing a YMC-Pack Diol-200 column (500×8.0 mm column) at a flow rate of 0.8 ml/min (0.1 M sodium phosphate, 0.15 M sodium chloride, pH 7.0). Protein elution was monitored at 220 nm.

**Figure 2:**
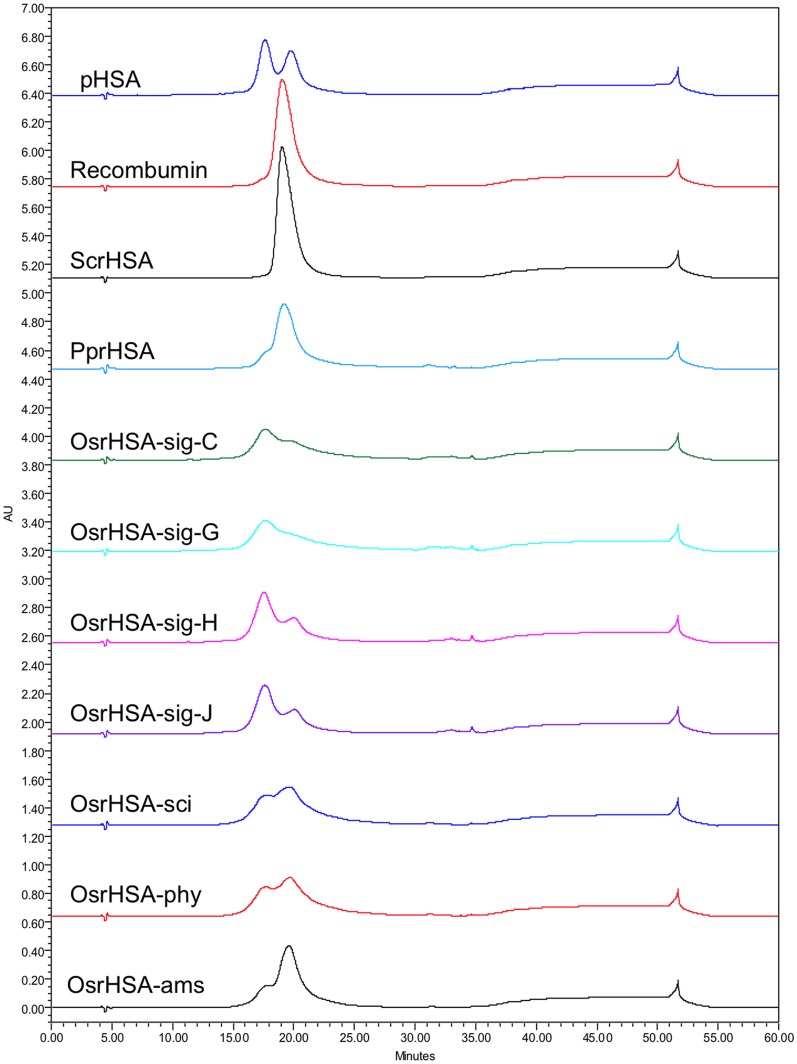
Representative chromatograms of pHSA and rHSA products obtained by RP-HPLC utilizing an Aquapore RP-300 column (C8, 7 µm, 220×2.1 mm i.d.) with protein elution was monitored at 220 nm.

SEC showed the main peak eluting at approximately 17.3–17.4 minutes for all samples with relative quantities of injected sample (calculated through integration of peak areas) for this peak ranging from 66.6% for OsrHSA-sig-C to 99.4% for ScrHSA (Figure 1, Table S1). Peaks of higher molecular weights (eluting between 12 and 16 minutes post-injection) were observed for pHSA, all OsrHSA samples, PprHSA and Recombumin (Figure 2, Table S1). OsrHSA samples generally showed a higher percentage of these dimers/oligomers and based on the quantity of higher molecular weight species present, Sigma-Aldrich supplied OsrHSA lots can be divided into two groups. The first group (OsrHSA-sig-C and OsrHSA-sig-G) had higher molecular weight peaks representing ∼16–20% of the injected samples whereas in the second group (OsrHSA-sig-H and OsrHSA-sig-J), these peaks represented only ∼8.3% of the injected samples (Figure 1, Table S1). Peaks eluting later than the main peak were observed for Sigma-Aldrich sourced material and PprHSA (Figure 1, Table S1) with elution times between 18.2 and 20.5 minutes potentially indicative of degradation fragments or protein contaminants. Further supporting this view is that the UV spectra of the main peak, the dimer and the lower MW products are almost identical, a good indication that they are structurally related (data not shown). The differences in the detection of small molecular species between SDS-PAGE and SEC may be attributed to sample preparation as SEC was run in non-reducing conditions whereas SDS-PAGE samples were prepared in DTT and boiled, potentially generating further small fragments.

RP-HPLC analysis showed pHSA to be heterogeneous (Figure 2) displaying two major peaks, 1 and 2, each of which has been previously shown to consist of several components with native HSA eluting as part of the more hydrophobic peak 2 [20]. RP-HPLC analysis of ScrHSA, Recombumin® and PprHSA (Figure 2) showed the major peak to be peak 2, suggesting that the majority of HSA was present in native forms. RP-HPLC of Sigma-Aldrich sourced OsrHSA showed the major peak of all lots to be peak 1, suggesting that the majority of Sigma-Aldrich sourced OsrHSA was present as modified forms (Figure 2). Similar to SEC analysis, the RP-HPLC profiles show the Sigma-Aldrich sourced OsrHSA lots can be categorized into two groups (OsrHSA-sig C/G and OsrHSA-sig H/J).

**Figure 3:**
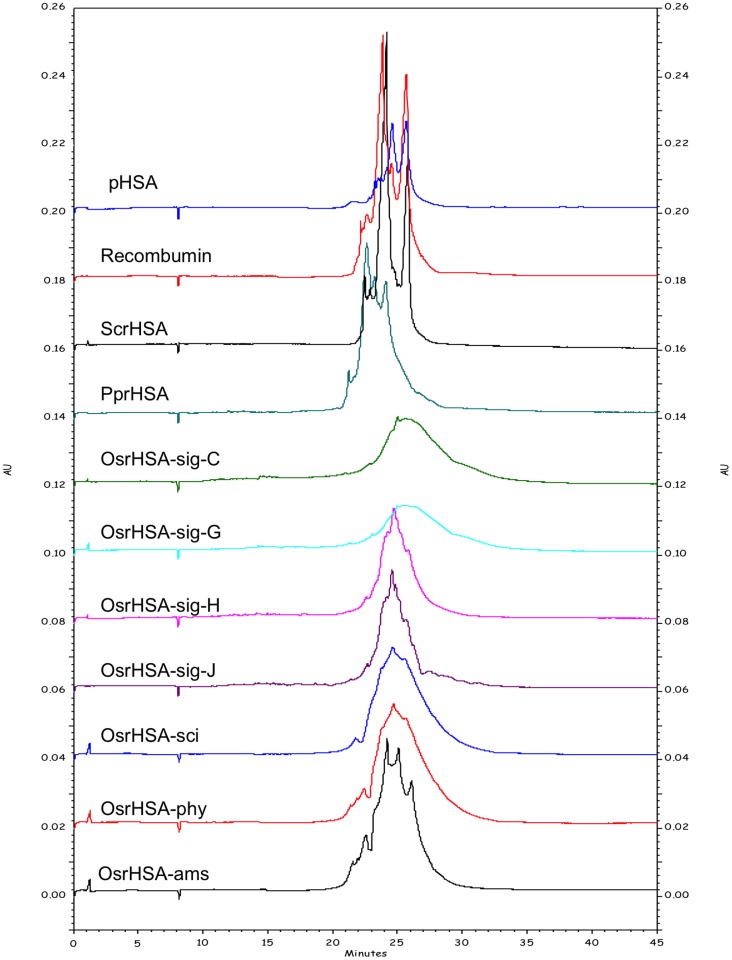
Representative electropherograms of pHSA and rHSA products utilizing a capillaries, with a 50 µm i.d. ×60 cm in length (50 cm effective length) using 20 mM disodium hydrogen phosphate containing 5 mM 1,4-diaminobutane and adjusted to pH 8.5. Protein elution was monitored at 220 nm.

CE analysis also showed significant heterogeneity of pHSA as previously reported [29], with three main groups of peaks between 23 and 26 minutes (Figure 3). Analysis of the OsrHSA products showed these products to have considerable supplier-to-supplier and lot-to-lot heterogeneity. For instance, Sigma-Aldrich sourced OsrHSA, CE analysis yielded a similar lot-to-lot variability to that observed with SEC and RP-HPLC. OsrHSA-sig H/J both display a broad peak centered at 25 minutes and OsrHSA-sig C/G both show a very broad flat peak centered at 26 minutes (Figure 3). These analyses clearly demonstrate the supplier-to-supplier and, more critically, lot-to-lot variability of rHSA expressed in *O. sativa*. Our previous mass spectrometric analyses of a single Sigma-Aldrich OsrHSA lot identified a higher incidence of hexose modification of lysine and arginine residues [3], which could account for the observed differences in sample profiles.

### Analysis of rHSA Products by Mass Spectrometry

To determine if differential glycation of lysine/arginine residues is responsible for the above observed supplier-to-supplier and lot-to-lot variability of OsrHSA, rHSA samples and pHSA were assessed by mass spectrometry. Analysis did not detect peptide sequences from yeast or plant protein contaminants from expression. Sequence coverage ranged from 95–97 percent (Table 1) with Mascot search results showing higher numbers of hexose modified lysine or arginine residues in all OsrHSA samples compared to rHSA expressed in yeast (Table 1). This analysis also showed that OsrHSA-sig-H and OsrHSA-sig-J have a reduced number of glycated residues compared to OsrHSA-sig-C and OsrHSA-sig-G; the identical grouping of lots observed with SEC, RP-HPLC and CE analysis. Oxidation of methionines was also detected on all samples and is not unexpected as methionine residues contribute to the protein's antioxidant activity and can be oxidized during sample preparation. To evaluate whether additional modifications were present, data were re-searched using Mascot's error-tolerant search algorithm. This yielded some novel peptide identifications, but they had low scores and few fragment ions matching theoretical values. Such identifications have a high probability of being false positive results and were therefore not considered further (data not shown).

**Table 1:** Mascot identifications of HSA hexose-modified peptides at lysine (K) or arginine (R) with scores>20.

Sample	Sequence Coverage %*	Number of Unique Hex(K) and Hex(R) Identified
pHSA	95	15
Recombumin®	96	8
ScrHSA	97	5
PprHSA	96	11
OsrHSA-sig-C	95	18
OsrHSA-sig-G	95	23
OsrHSA-sig-H	96	13
OsrHSA-sig-J	95	13
OsrHSA-sci	95	23
OsrHSA-phy	95	21
OsrHSA-ams	95	17

* Excluding leader sequence.

**Figure 4:**
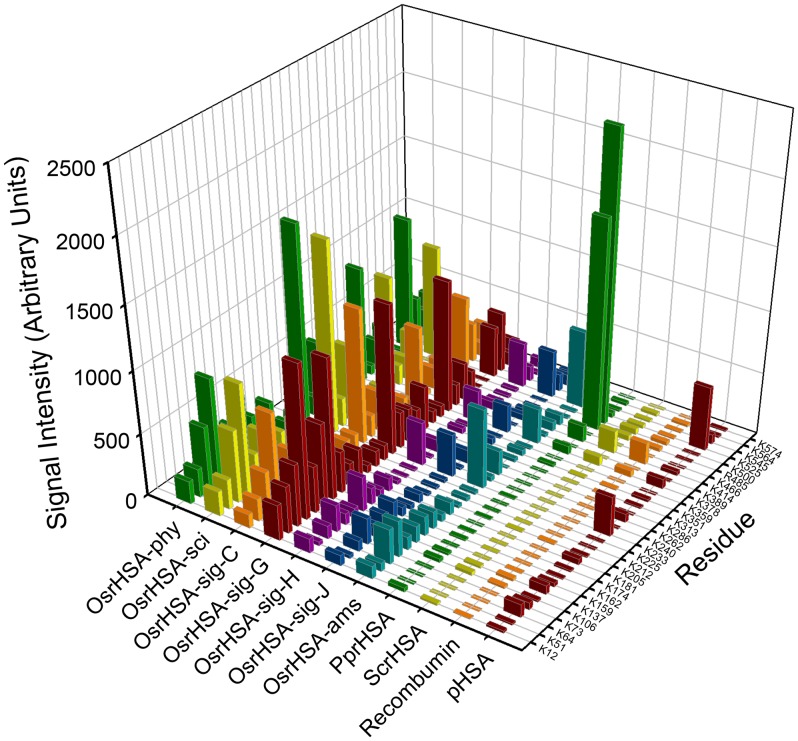
Signal intensity (arbitrary units) of peptides containing a hexose modified K/R.

To examine the hexose glycation in greater detail, the LC-MS data were further analyzed with Progenesis LC-MS software, which performed relative quantification of peptides between samples based on normalized ion abundance. For this quantification, peptides identified in any sample by Mascot were assigned a quantitative value in all samples by Progenesis, which aligns all the LC-MS chromatograms and compares peaks that have the same accurate mass and retention time [30]. Comparison of the peak intensities of specific glycopeptides yields estimates of the relative prevalence of glycation at specific residues between samples. Also, the sum of the peak intensities of all glycopeptides can be compared between samples as an estimate of relative overall prevalence of glycation. The specific signal intensities of peptides containing modified K/R residues are displayed in Figure 4, in which peptides are annotated by the position of modified residue (tabulated data available as Table S2). Although OsrHSA samples were glycated at many of the same lysine residues, the relative abundance of modification at specific residues varied greatly between samples and lots. This is best illustrated by residues K73, 233 and 378 where OsrHSA-ams, sig-H and sig-J show a low abundance of modified lysine residues compared to OsrHSA-sig-C, sig-G, phy and sci, but high abundance relative to pHSA, PprHSA, ScrHSA and Recombumin. Utilizing a 2.5 Å crystal structure of HSA by Sugio and coworkers [31], most identified glycated residues were on the surface of the protein with the exception of R485 and K525 which are buried or partially buried within the protein.

The summed intensity of all glycopeptides from the various rHSAs is shown in Table S3 and serves as an estimate of the prevalence of glycated isoforms relative to pHSA. Progenesis LC-MS analysis clearly illustrates the differences in glycation between rHSA expressed in rice and yeast expression systems as well as the supplier-to-supplier and lot-to-lot variability of OsrHSA.

Glycation occurs via a slow non-enzymatic Maillard reaction in which residues with free amine groups are modified with sugars [13]. Detection of pHSA with hexose-modified lysine and arginine residues is not unexpected as up to 10% of pHSA is glycated in healthy individuals and up to 30% in individuals with hyperglycaemia [1]. *In vivo* glycation of pHSA occurs over the long (26–31 day) circulation lifetime [1] of the protein whereas *in vitro* glycation of lysine and arginine requires elevated temperature and sugar concentrations as well as a time scale on the order of days or weeks [14], [32].

Although the exact method of production and purification of yeast-expressed rHSA used in these studies is unknown to us, published accounts of the expression of rHSA in *P. pastoris* or *S. cerevisiae* indicate that the recombinant protein is secreted during expression [33], [34]. Subsequently, the sugars in the growth media (up to 2% glucose) could provide an environment suitable for the glycation of the secreted protein. It has been suggested that the glycation mechanisms in plants may involve light/dark cycles or light stress to the plants [35] and glycation of plant proteins is not unexpected as higher order plants possess homologues of animal enzymes that repair early glycation adducts [35]. OsrHSA used in these experiments is expressed in the endosperm of *O. sativa* [36] which contains up to 19 mg of glucose per g total weight [37]. The presence of this monosaccharide may allow for the glycation of the protein over the growth period of the plant (approximately 30 days for grain ripening [38]) and variations in growth conditions of the plant may account for the lot-to-lot variability of Sigma-Aldrich supplied OsrHSA.

Patterns of OsrHSA glycation can partially explain the results from SEC, RP-HPLC and CE analysis. For example, the high degree of glycation in OsrHSA-sig-C and G correlates with the greater relative amounts of high molecular species observed by SEC for these samples. Glycation has been implicated in protein aggregation and previous studies have shown that glycation can increase the content of non-monomeric forms of bovine serum albumin (BSA) [14] potentially through lysine-lysine crosslinking as a result of the Maillard reaction [39]. Lysine-lysine crosslinking may also explain the presence of DTT resistant aggregates observed with the SDS-PAGE (Figure S1).

Glycation also alters the hydrophobicity/hydrophilicity balance of proteins by increasing the net negative surface charge of a protein, which would alter both RP-HPLC and CE profiles. OsrHSA-sci and OsrHSA-phy with a greater number of glycated arginine and lysine residues (and overall greater degree of glycation of those residues when compared to OsrHSA-ams) show chromatograms with a more predominant hydrophilic profile than those of OsrHSA-ams. Interestingly, Sigma-Aldrich supplied OsrHSA samples do not display a similar pattern between glycation and the prevalence of more hydrophilic peaks, with all lots showing predominance of peak 1 regardless of the prevalence of glycation. This suggests the presence of additional modifications on Sigma-Aldrich OsrHSA samples and/or bound ligands altering the chromatographic profiles. One possible explanation is the presence of oxidized methionines which can also lead to earlier eluting peaks for RP-HPLC. Future investigation to explain the RP-HPLC results for Sigma-Aldrich OsrHSA could provide data for differentiating the contribution of glycation and/or oxidation for these varying elution profiles.

### Analysis of rHSA Thermal Stability

**Figure 5:**
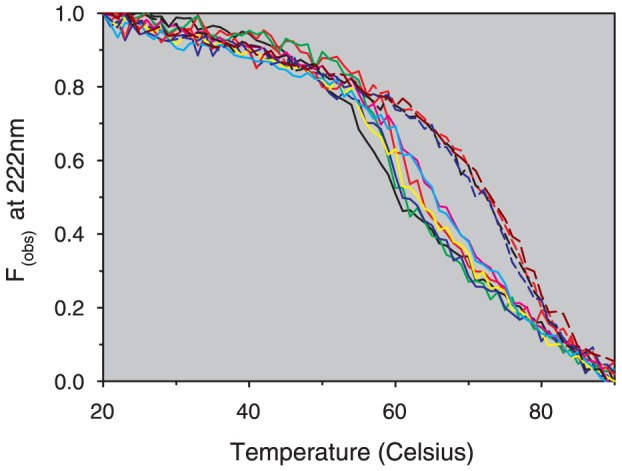
Fractional change in ellipticity at 222 nm (Fobs) with temperature for serum albumins as monitored by far-UV circular dichroism with a Jasco 815 spectropolarimeter, pHSA (black), Recombumin (red), ScrHSA (green), PprHSA(blue), OsrHSA-ams (yellow), OsrHSA-sci (pink), OsrHSA-phy (cyan), OsrHSA-sig-C (dashed red), OsrHSA-sig-G (dashed black), OsrHSA-sig-H (dashed blue) and OsrHSA-sig-J (dashed red). Each spectrum represents the mean of at least 3 separate experiments.

The presence of glycated residues or bound FAs is significant as they can dramatically alter the biophysical properties of the protein. Thermal stability is of particular interest, as a number of studies have shown a positive correlation between structural stability of protein therapeutics and efficacy and/or *in vivo* circulation lifetimes [40]–[43]. Analysis by far U/V CD clearly shows that as supplied, OsrHSA-sig-C, G, H and J have noticeably higher thermal stability than other samples. An approximate increase of 10–12°C was required to lose 50 percent of their alpha-helical content when compared to pHSA or the remaining rHSA samples (Figure 5).

**Figure 6:**
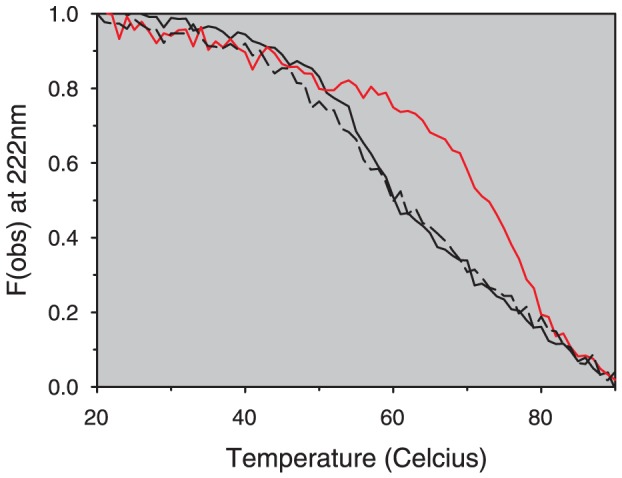
Fractional change in ellipticity at 222 nm (Fobs) with temperature for pHSA (black), OsrHSA-sig-C (red) and dfOsrHSA-sig-c (Black dashed) as monitored by far-UV circular dichroism with a Jasco 815 spectropolarimeter. Each spectrum represents the mean of at least 3 separate experiments.

Although both glycation and bound FAs have been shown to alter the thermal stability of albumin [14], [32], [44]–[47] we had previously postulated, through comparisons of fatted and defatted BSA, that bound FAs (and not the extensive glycation of Sigma-Aldrich OsrHSA) were responsible for the improved thermal stability [3]. To definitively determine if either bound FAs or extensive glycation were responsible for the improved thermal stability of Sigma-Aldrich OsrHSA lots, OsrHSA-sig-C (which displays extensive glycation and improved thermal stability) was defatted and its thermal stability subsequently assessed. SDS-PAGE analysis showed no significant changes in major band patterns between OsrHSA-sig-C and dfOsrHSA-sig-C and analysis with SEC confirmed the defatting procedure did not result in the generation of additional dimers or larger aggregates for dfOsrHSA-sig-C (data not shown). Thermal stability analysis of this defatted sample showed a reduction in stability to a level similar to that of pHSA (Figure 6) implying that bound FAs and not lysine/arginine glycation are responsible for the improved stability of OsrHSA and confirming our previous results. These results were confirmed with DSC analysis (Figure S2).

Sodium-octanoate and *N*-acetyl-_L_-tryptophanate have been used as thermal stabilizers for commercial preparations of pHSA during pasteurization, yielding an approximate 7°C increase in T_m_ as measured by DSC [48]. Alternatively, rice seeds have been shown to contain approximately three percent oil by dry weight [49] and prior studies have identified long chain lipids lysophosphatidylcholine, lysophosphatidylethanolamine and phosphatidylcholine associated with the OsrHSA expressed in rice endosperm [50]. Our DSC studies show a nearly 24°C improvement of OsrHSA-sig-C over pHSA (Figure S2). Regardless of the source of the stabilizing FAs, their presence in multiple lots of Sigma-Aldrich supplied OsrHSA suggests their occurrence is a consequence of the production or purification process.

### Analysis of rHSA Structure

Glycation of albumin has been shown to reduce α-helical content with a resultant increase in β-sheet content as well as changes to tertiary structure [12]–[14], [44], [46]. This is critical as changes to therapeutic protein structure have been implicated in adverse reactions and/or reduced efficacy [51], [52]. Far U/V CD analysis of secondary structure showed pHSA and all rHSA samples had high levels of alpha-helical content (Table 2) in agreement with previous studies [3], [45]. Minor differences in alpha-helical content between samples were noted, with Recombumin and ScrHSA having slightly higher levels than pHSA, and OsrHSA-sig-C, G, H and J having slightly lower levels; similar to previous trends observed with OsrHSA [3]. Removal of FAs from OsrHSA-sig-C showed no change in secondary structural content (Table 2). It should also be noted that oxidation of albumin has been shown to have minimal effects on secondary structure.

**Table 2:** Calculated secondary structural content from far U/V CD analysis and intrinsic fluorescence analysis of pHSA and various rHSA samples.

Sample	% Alpha helix	% Beta Structure	% Unordered	Max. Fluorescence Intensity (RFU)	λ_em_max (nm)
pHSA	67.0+/−0	13.0+/−0	20.3+/−0.6	631.6+/−21.8	343.2+/−1.9
Recombumin®	69.3+/−0.6	12.0+/−0	19.7+/−0.6	685.4+/−22.0	346.3+/−1.2
ScrHSA	69.0+/−1.0	12.7+/−0.6	18.7+/−0.6	701.6+/−27.9	346.4+/−1.1
PprHSA	67.7+/−0.6	13.3+/−0.6	20.0+/−0	520.4+/−8.7	343.2+/−1.9
OsrHSA-sig-C	65.3+/−0.6	15.7+/−0.6	20.0+/−0	584.9+/−12.6	339.6+/−1.5
dfOsrHSA-sig-C	66.0+/−1.0	14.0+/−1.0	20.3+/−0.6	590.4+/−21.1	342.8+/−1.9
OsrHSA-sig-G	66.7+/−0.6	14.3+/−1.5	19.0+/−0	565.7+/−17.8	338.8+/−2.1
OsrHSA-sig-H	64.7+/−0.6	15.7+/−0.6	20.3+/−0.6	627.8+/−10.6	340.7+/−1.0
OsrHSA-sig-J	65.0+/−1.7	14.3+/−1.5	20.3+/−0.6	617.6+/−16.0	340.7+/−1.0
OsrHSA-sci	68.3+/−1.5	12.7+/−0.6	19.3+/−1.2	566.7+/−3.9	345.3+/−2.0
OsrHSA-phy	67.0+/−1.7	12.7+/−1.5	19.7+/−0.6	555.2+/−18.7	344.4+/−1.4
OsrHSA-ams	66.0+/−0.0	13.3+/−0.6	20.3+/−0.6	594.5+/−25.4	345.0+/−1.3

Mean values ± standard deviation for at least 3 separate experiments.

Glycation and the presence of bound FAs can also alter the fluorescence of albumin. Glycation of arginine/lysine residues can result in changes to quantum yield (fluorescence intensity) though tertiary structural rearrangements and subsequent quenching of the Trp214 by newly adjacent residues [12], [32], [53]. The presence of FAs may alter the emission wavelength (λ_em_max) by changing the micro-environment surrounding Trp214 [16], [54]. Both Recombumin and ScrHSA showed enhanced relative tryptophan fluorescence intensity when compared to pHSA. Whereas PprHSA, OsrHSA-sci, OsrHSA-phy, OsrHSA-ams, OsrHSA-sig-C and OsrHSA-sig-C showed reduced relative tryptophan fluorescence intensity compared to pHSA with a positive correlation between the number and intensity of peptides containing glycated residues and a reduction of fluorescence intensity was observed (Table 2). The altered fluorescence intensity for PprHSA, OsrHSA-sci, OsrHSA-phy, OsrHSA-ams, OsrHSA-sig-C and OsrHSA-sig-C suggest alterations in tertiary structure for these samples. Additionally, a blue shift in λ_em_max was observed for all Sigma-Aldrich sourced OsrHSA samples (Table 2) as compared to pHSA. Defatting OsrHSA-sig-C resulted in a red shift of λ_em_max suggesting that FAs were bound near Trp214 and were responsible for the original λ_em_max value but did not increase the intrinsic fluorescence intensity of the sample, implying the extensive glycation of OsrHSA lots is responsible for alterations to the proteins' tertiary structure (Table 2). Although previous studies have shown that oxidation of HSA can alter tertiary structure [55], the presence of an oxidized methionine on all samples suggests that the differences in tertiary structure are due to the presence of glycated residues.

### Analysis of rHSA Drug Binding

**Figure 7:**
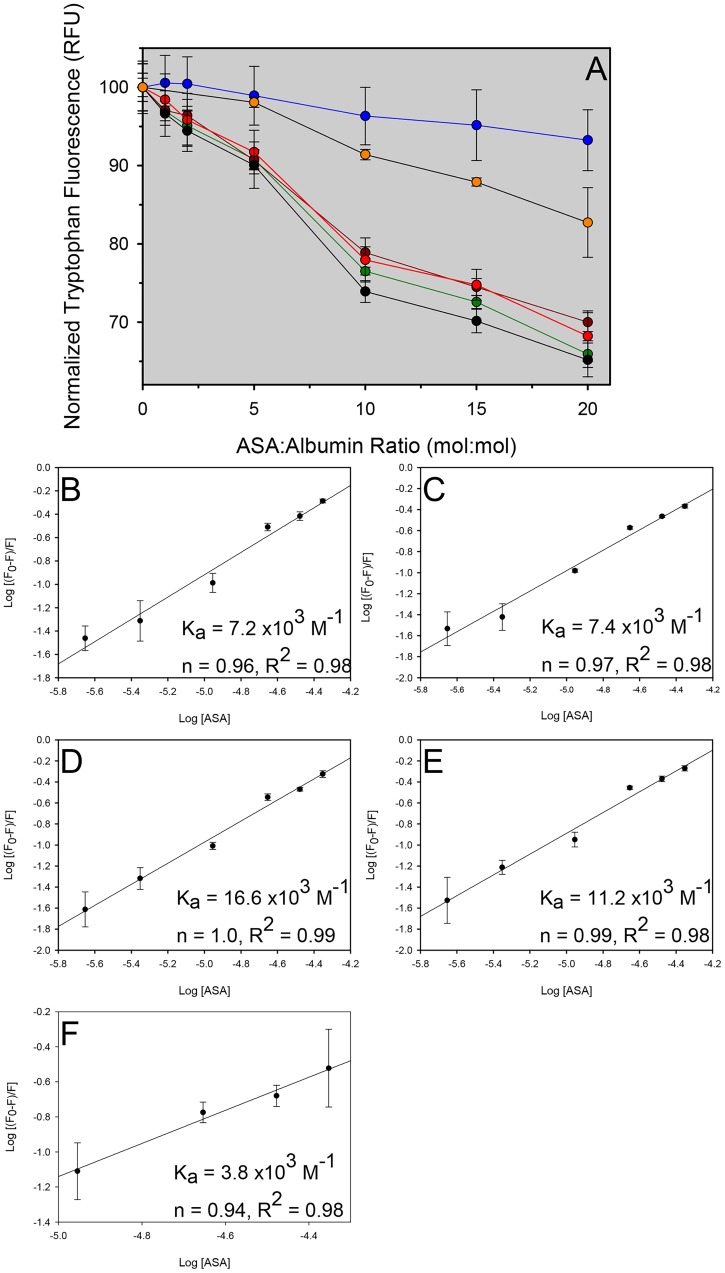
(A) Quenching of tryptophan fluorescence of six albumin samples in the presence of ASA OsrHSA-sig-C (blue), ScrHSA (green), PprHSA (dark red), Recombumin (red), pHSA (black), dfOsrHSA-sig-C (orange). Each plot represents the mean of at least 3 separate experiments with error bars representing standard deviations. Plots of log [(F_0_ – F)/F] vs log [ASA] and calculated *K_a_* and n values for PprHSA (B), ScrHSA (C), Recombumin® (D), pHSA (E) and dfOsrHSA-sig-C (F).

Bound FAs or glycation, in addition to altering structure and stability, can also alter drug binding to albumin [15], [27], [56]. Clinical doses of albumin can be as high as 125 g [6], representing approximately 50–60 percent of circulating albumin (at an albumin concentration of 30–50 g/L of blood [5], [13] and a typical adult circulating blood volume of 5L). Since albumin plays a critical role in drug distribution and bioavailability, it is important to assess the drug-binding capacity of heavily glycated, thermally stable OsrHSA. To this end, OsrHSA-sig-C was compared to pHSA and rHSA expressed in yeast. ASA has been shown to predominantly bind albumin at Sudlow I site, a hydrophobic pocket located in subdomain IIA [57]. It should be noted that Anraku and coworkers [55] have shown that oxidation of can alter drug binding to albumin at Sudlow II site but does not affect drug binding at Sudlow I site. Binding was monitored by drug-induced quenching of intrinsic fluorescence as described previously [26], [27]. All HSA samples tested, except OsrHSA-Sig-C, showed similar quenching of intrinsic fluorescence in the presence of increasing concentrations of ASA (Figure 7a). Plotting log [(F_0_ – F)/F] vs log [ASA], where F_0_ is initial fluorescence and F is measured fluorescence in the presence of a quencher, the binding constant (*K*_a_) and number of bound binding sites (n) per albumin were estimated (Figure 7b–e). It was not possible to calculate n or *K*_a_ for OsrHSA due to a lack of ASA-induced tryptophan fluorescence quenching.

Defatting of OsrHSA-sig-C increased the ASA-induced tryptophan fluorescence quenching, but not to levels similar to those of the other rHSAs or pHSA (Figure 7a,f). This suggests that bound FAs are only partially responsible for reduced ASA binding and that the extensive glycation of OsrHSA sig-C also contributes to the reduction of ASA binding. Similar results were observed by Baraka-Vidot and coworkers in which glycation reduced binding of both Sudlow I and II site-specific drugs [27]. Glycation of lysine and arginine residues has been shown to alter local secondary structure [14], [58] and glycation of residues on the alpha helices or loops bridging alpha helices defining the Sudlow I and II sites could modify their structure resulting in altered drug binding. Glycated lysine residues were detected (K181 and K240) on two alpha helices that help define the hydrophobic pocket of the Sudlow I site as well as two on loops connecting alpha helices (K205 and K225) within the site. The Sudlow II site also shows similar modifications with glycated residues (K389 and K414) on two alpha helices that define the binding site. Disruption of these helices containing these glycated residues may also partially account for the reduction in alpha helical secondary structure content observed with far U/V CD.

## Conclusions

In this study, we present an extensive analysis of rHSA expressed in Asian rice (*Oryza sativa*) from a number of suppliers, as well as multiple lots from a single supplier. All OsrHSA samples showed elevated levels of arginine and lysine hexose glycation compared to rHSA expressed in yeast, suggesting that the extensive glycation of the recombinant proteins is a by-product of either the expression system or purification process and not a random occurrence. The extensive glycation of OsrHSA observed could have implications for therapeutic use of the protein since plant-specific sugars such as α-1,3- fucose and β-1,2-xylose have been associated with adverse immune reactions [59].

Critically, we also observed lot-to-lot variability in glycation for four lots of OsrHSA obtained from Sigma-Aldrich with two lots showing a higher number and abundance of glycated peptides identified by trypsin digestion and LC-MS analyses. As observed here, as well as noted in other studies, the level of protein glycation can significantly impact biophysical properties of HSA such as structure and drug binding, and may have implications regarding the utility of these proteins in particular studies. For instance HSA has been used as a model protein for numerous studies investigating protein/drug binding and protein/nano-particle interactions. Lot-to-lot variability may make any conclusions drawn from these or future studies using OsrHSA difficult to interpret. Furthermore, altered drug binding could be significant for the future consideration of heavily glycated OsrHSA with regards to individuals receiving large doses of recombinant albumin expressed in *O. sativa*.

We also demonstrated that Sigma-Aldrich sourced OsrHSAs have dramatically improved thermal stability, due to the presence of FAs, when compared to other OsrHSAs, which could impact their function as biotherapeutics. However, the source and composition of these FAs or the potential for lot-to-lot variability in their composition have yet to be determined.

## Supporting Information

Figure S1 **SDS-PAGE and SYPRO ruby staining of pHSA and various rHSAs. Lane 1: ScrHSA; lane 2: OsrHSA-sig-C; lane 3: OsrHSA-sig-G; lane 4: OsrHSA-sig-H; lane 5: OsrHSA-sig-J; lane 6: PprHSA; lane 7: benchmark protein ladder (arrows indicate 50 and 20 kDa bands); lane 8: Recombumin; lane 9: pHSA; lane10: OsrHSA-sci; lane 11: OsrHSA-phy; lane 12: OsrHSA-ams.**
(PDF)

Figure S2 **Differential scanning calorimetry analysis for the six albumin samples assayed [ScrHSA (green, Tm°C = 62.0+/−0.1), OsrHSA (blue, Tm°C = 81.4+/−0.2), PprHSA (dark red, Tm°C = 61.3+/−0.3), Recombumin (red, Tm°C = 63.6+/−0), pHSA (black, Tm°C = 57.5+/−0.1), dfOsrHSA-sig-C (orange, Tm°C = 56.0+/−0.3)].** Each plot represents the mean of at least 3 separate experiments.
(EPS)

Table S1 **Peak retention times and integrated area for pHSA and various rHSAs from SEC analysis.**
(DOCX)

Table S2 **Signal intensity (arbitrary units) of peptides containing a hexose modified K/R from pHSA and various rHSAs.**
(DOCX)

Table S3 **Summed signal intensity of all peptides containing a hexose modified K/R (relative to pHSA).**
(DOCX)
