# Supplementary figures and images for: Determination of Supplier-to-Supplier and Lot-to-Lot Variability in Glycation of Recombinant Human Serum Albumin Expressed in Oryza sativa
Source: PLoS One. 2014 Oct 9;9(10):e109893. doi: 10.1371/journal.pone.0109893 (PMC4192584; doi:10.1371/journal.pone.0109893)

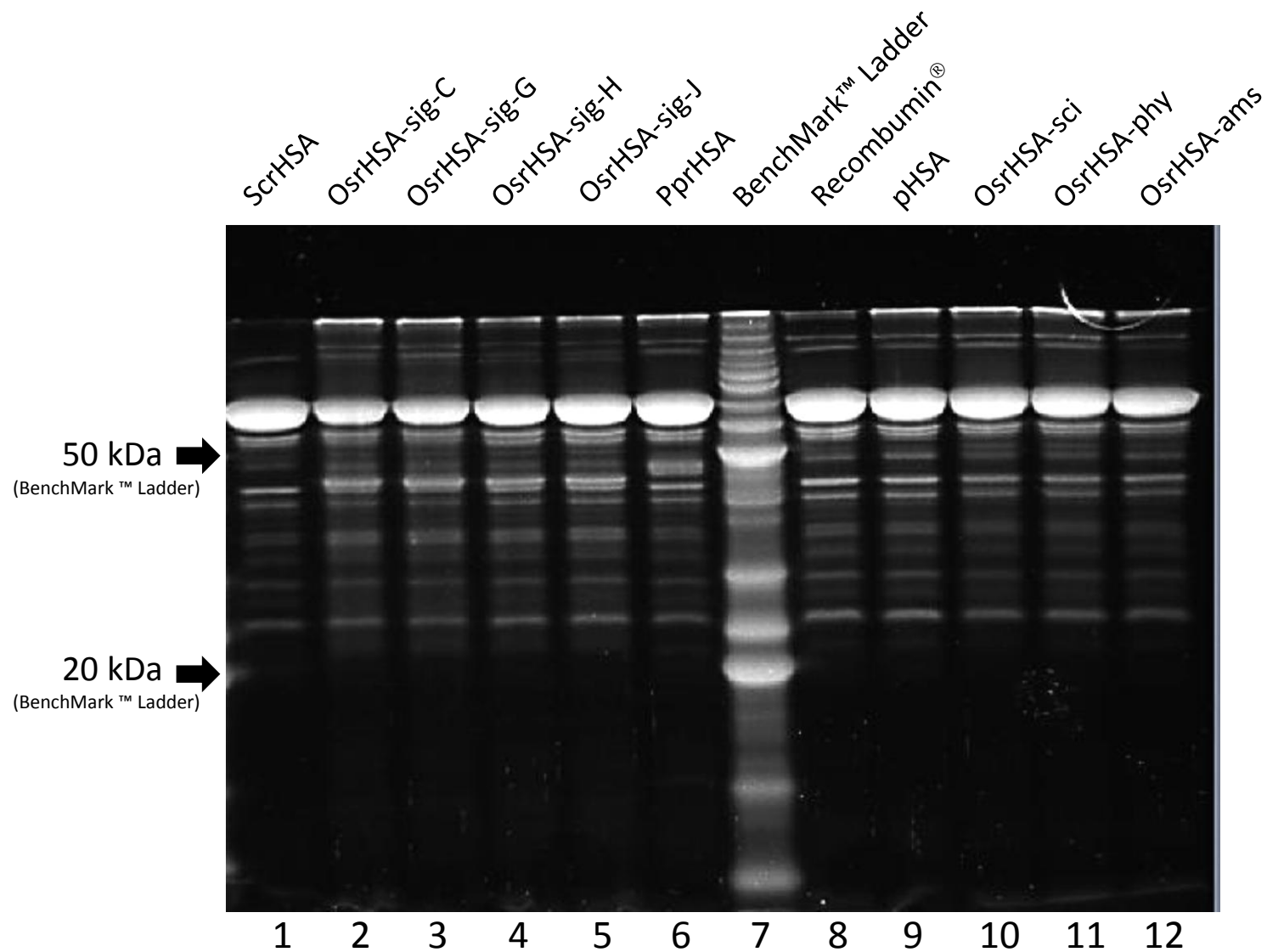

Supplement: Figure S1 — SDS-PAGE and SYPRO ruby staining of pHSA and various rHSAs. Lane 1: ScrHSA; lane 2: OsrHSA-sig-C; lane 3: OsrHSA-sig-G; lane 4: OsrHSA-sig-H; lane 5: OsrHSA-sig-J; lane 6: PprHSA; lane 7: benchmark protein ladder (arrows indicate 50 and 20 kDa bands); lane 8: Recombumin; lane 9: pHSA; lane10: OsrHSA-sci; lane 11: OsrHSA-phy; lane 12: OsrHSA-ams. (PDF) [file pone.0109893.s001.pdf]
