# Supplementary material for: Determination of Supplier-to-Supplier and Lot-to-Lot Variability in Glycation of Recombinant Human Serum Albumin Expressed in Oryza sativa
Source: PLoS One. 2014 Oct 9;9(10):e109893. doi: 10.1371/journal.pone.0109893 (PMC4192584; doi:10.1371/journal.pone.0109893)
Supplement: Table S1 — Peak retention times and integrated area for pHSA and various rHSAs from SEC analysis. (DOCX) [file pone.0109893.s003.docx]

| **Sample/Peak#** | **Peak retention time (min)/Percent Area of Chromatogram** | | | | | | | |
| --- | --- | --- | --- | --- | --- | --- | --- | --- |
|  | **1** | **2** | **3** | **4** | **5** | **6** | **7** | **8** |
| pHSA | 13.1/0.1 | 13.8/0.6 | 14.3/2.5 | 15.4/12.2 | 17.4/84.6 |  |  |  |
| Recombumin |  |  | 14.3/0.2 | 15.4/4 | 17.4/95.9 |  |  |  |
| ScrHSA |  |  |  | 15.3/0.6 | 17.4/99.4 |  |  |  |
| PprHSA |  |  | 14.4/1.6 | 15.4/9.36 | 17.4/84.5 | 18.2/4.3 |  |  |
| OsrHSA-sig-C | 13.0/0.6 |  | 14.4/5.1 | 15.3/14.9 | 17.3/66.6 | 18.4/8.5 | 19.8/1.7 | 20.4/2.5 |
| OsrHSA-sig-G | 13.0/1.0 |  | 14.4/3.1 | 15.3/12.1 | 17.3/69.9 | 18.4/9.5 | 19.7/1.9 | 20.4/2.7 |
| OsrHSA-sig-H | 12.9/1.2 |  | 14.4/0.9 | 15.4/6.2 | 17.3/80.1 | 18.4/7.7 | 19.8/1.4 | 20.5/1.9 |
| OsrHSA-sig-J | 12.9/1.2 |  | 14.4/0.9 | 15.4/6.2 | 17.3/80.6 | 18.4/7.7 | 19.8/1.4 | 20.5/1.9 |
| OsrHSA-sci | 12.9/0.5 |  | 14.3/3.7 | 15.4/12.7 | 17.4/83.1 |  |  |  |
| OsrHSA-phy | 12.9/0.7 | 13.8/0.9 | 14.3/3.3 | 15.4/13.1 | 17.4/82.0 |  |  |  |
| OsrHSA-ams | 12.9/0.3 | 13.7/0.2 | 14.4/1.6 | 15.4/9.1 | 17.4/88.8 |  |  |  |
