# Supplementary material for: Determination of Supplier-to-Supplier and Lot-to-Lot Variability in Glycation of Recombinant Human Serum Albumin Expressed in Oryza sativa
Source: PLoS One. 2014 Oct 9;9(10):e109893. doi: 10.1371/journal.pone.0109893 (PMC4192584; doi:10.1371/journal.pone.0109893)
Supplement: Table S2 — Signal intensity (arbitrary units) of peptides containing a hexose modified K/R from pHSA and various rHSAs. (DOCX) [file pone.0109893.s004.docx]

|  | pHSA | Recombumin | ScrHSA | PprHSA | OsrHSA-sig-C | OsrHSA-sig-G | OsrHSA-sig-H | OsrHSA-sig-J | OsrHSA-sci | OsrHSA-phy | OsrHSA-ams |
| --- | --- | --- | --- | --- | --- | --- | --- | --- | --- | --- | --- |
| K12 | 18.4 | 7.0 | 16.6 | 33.4 | 109.1 | 285.8 | 116.4 | 84.6 | 197.1 | 183.0 | 101.4 |
| K51 | 13.8 | 0.0 | 0.0 | 0.2 | 180.1 | 380.2 | 33.4 | 27.4 | 238.6 | 228.7 | 67.3 |
| K64 | 104.1 | 0.2 | 0.1 | 0.4 | 342.5 | 500.3 | 104.4 | 84.7 | 583.1 | 519.0 | 281.2 |
| K73 | 56.4 | 0.5 | 2.4 | 2.9 | 765.7 | 1241.3 | 215.9 | 210.4 | 906.1 | 857.4 | 291.1 |
| K106 | 15.4 | 17.8 | 26.2 | 30.3 | 168.3 | 375.2 | 92.1 | 100.5 | 286.8 | 291.1 | 102.6 |
| K137 | 71.3 | 22.7 | 17.3 | 19.6 | 359.6 | 665.8 | 79.4 | 127.9 | 424.6 | 368.6 | 132.4 |
| K159 | 32.9 | 5.0 | 1.9 | 3.7 | 526.1 | 1136.3 | 277.6 | 174.2 | 394.0 | 560.5 | 153.2 |
| K162 | 8.5 | 2.2 | 4.4 | 13.0 | 329.8 | 333.6 | 133.4 | 74.1 | 314.0 | 347.3 | 137.7 |
| K174 | 14.4 | 2.8 | 3.6 | 8.1 | 211.6 | 184.9 | 65.1 | 37.9 | 205.6 | 209.4 | 58.0 |
| K181 | 47.6 | 1.1 | 0.8 | 0.6 | 287.7 | 245.7 | 103.6 | 69.8 | 294.0 | 354.4 | 100.9 |
| K205 | 9.7 | 10.3 | 8.6 | 6.9 | 49.4 | 61.5 | 30.5 | 21.6 | 61.5 | 51.1 | 25.0 |
| K212 | 3.4 | 1.0 | 3.6 | 4.7 | 117.7 | 126.1 | 9.1 | 20.8 | 72.6 | 86.5 | 30.9 |
| K225 | 4.4 | 0.9 | 2.6 | 3.2 | 116.9 | 121.2 | 16.5 | 26.6 | 83.4 | 105.5 | 22.9 |
| K233 | 320.9 | 0.0 | 0.0 | 7.7 | 1092.5 | 1211.0 | 350.0 | 343.6 | 1548.3 | 1594.9 | 643.2 |
| K240 | 3.1 | 0.8 | 1.7 | 1.5 | 174.7 | 292.2 | 16.7 | 20.6 | 227.5 | 156.4 | 39.2 |
| K262 | 67.9 | 2.1 | 2.0 | 2.1 | 325.3 | 270.9 | 100.5 | 108.7 | 615.3 | 549.5 | 197.2 |
| K286 | 0.0 | 0.0 | 0.0 | 0.0 | 20.9 | 210.3 | 0.0 | 0.3 | 21.0 | 33.8 | 2.0 |
| K313 | 12.3 | 6.5 | 3.5 | 2.5 | 56.1 | 350.9 | 5.1 | 10.5 | 137.0 | 142.0 | 29.2 |
| K351 | 22.0 | 12.1 | 10.5 | 21.2 | 120.4 | 97.2 | 43.9 | 50.5 | 84.2 | 117.3 | 45.8 |
| K359 | 0.3 | 0.5 | 0.0 | 0.1 | 52.3 | 92.1 | 17.2 | 10.9 | 73.0 | 57.5 | 13.5 |
| K378 | 77.7 | 52.1 | 72.3 | 67.9 | 608.9 | 1069.5 | 255.9 | 225.9 | 935.8 | 932.1 | 285.8 |
| K389 | 4.2 | 0.0 | 0.0 | 0.0 | 34.2 | 171.3 | 0.0 | 4.3 | 161.1 | 74.3 | 24.0 |
| K414 | 32.2 | 174.7 | 181.7 | 131.9 | 175.9 | 300.4 | 65.1 | 50.3 | 184.7 | 238.8 | 72.1 |
| K466 | 0.4 | 0.0 | 0.0 | 0.0 | 189.6 | 188.3 | 24.2 | 32.3 | 341.2 | 311.1 | 81.7 |
| R485 | 4.7 | 29.0 | 41.0 | 1693.9 | 2.2 | 6.2 | 19.4 | 5.7 | 2.1 | 13.1 | 12.2 |
| K500 | 4.7 | 31.0 | 46.2 | 2319.6 | 2.2 | 6.2 | 22.3 | 5.9 | 2.1 | 17.1 | 12.9 |
| K525 | 520.5 | 11.8 | 25.0 | 39.1 | 575.8 | 409.6 | 361.8 | 379.7 | 915.5 | 1071.3 | 629.2 |
| K545 | 77.6 | 11.1 | 15.0 | 15.5 | 314.3 | 485.9 | 120.2 | 138.1 | 367.4 | 368.9 | 165.6 |
| K564 | 16.9 | 21.0 | 23.4 | 24.2 | 109.6 | 109.9 | 25.9 | 89.3 | 149.7 | 227.1 | 38.8 |
| K574 | 11.7 | 0.7 | 0.0 | 0.0 | 216.5 | 168.8 | 79.4 | 62.7 | 351.4 | 312.5 | 70.1 |
