# Supplementary material for: Determination of Supplier-to-Supplier and Lot-to-Lot Variability in Glycation of Recombinant Human Serum Albumin Expressed in Oryza sativa
Source: PLoS One. 2014 Oct 9;9(10):e109893. doi: 10.1371/journal.pone.0109893 (PMC4192584; doi:10.1371/journal.pone.0109893)
Supplement: Table S3 — Summed signal intensity of all peptides containing a hexose modified K/R (relative to pHSA). (DOCX) [file pone.0109893.s005.docx]

| **Sample** | **Relative total signal intensity** |
| --- | --- |
| pHSA | 1.0 |
| Recombumin^®^ | 0.3 |
| ScrHSA | 0.3 |
| PprHSA | 2.8 |
| OsrHSA-sig-C | 4.8 |
| OsrHSA-sig-G | 7.0 |
| OsrHSA-sig-H | 1.8 |
| OsrHSA-sig-J | 1.6 |
| OsrHSA-sci | 6.5 |
| OsrHSA-phy | 6.6 |
| OsrHSA-ams | 2.5 |
